# Supplementary figures and images for: Genome-wide chromatin contacts of super-enhancer-associated lncRNA identify LINC01013 as a regulator of fibrosis in the aortic valve
Source: PLoS Genet. 2022 Jan 18;18(1):e1010010. doi: 10.1371/journal.pgen.1010010 (PMC8797204; doi:10.1371/journal.pgen.1010010)

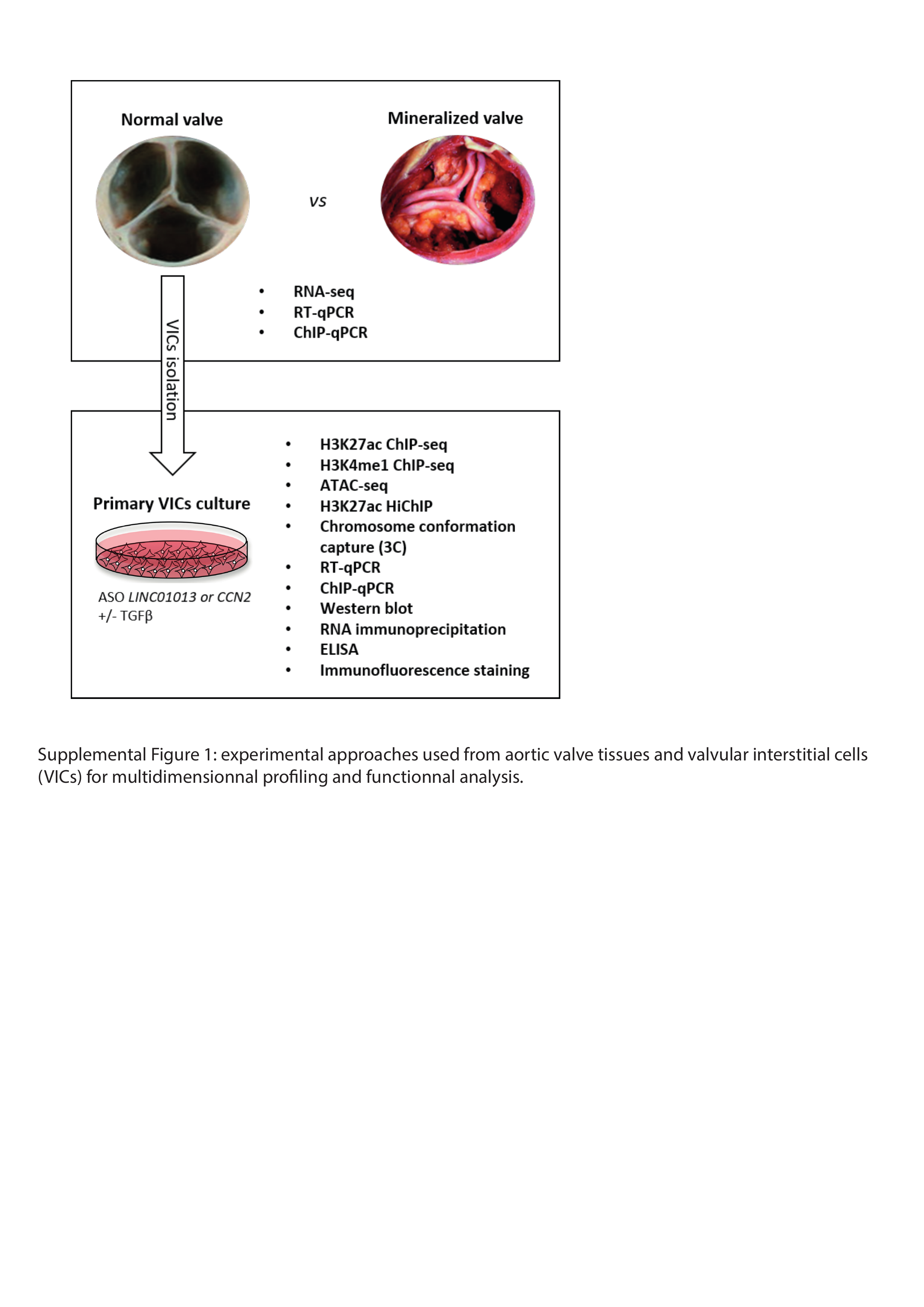

Supplement: S1 Fig — (TIFF) [file pgen.1010010.s001.tiff]

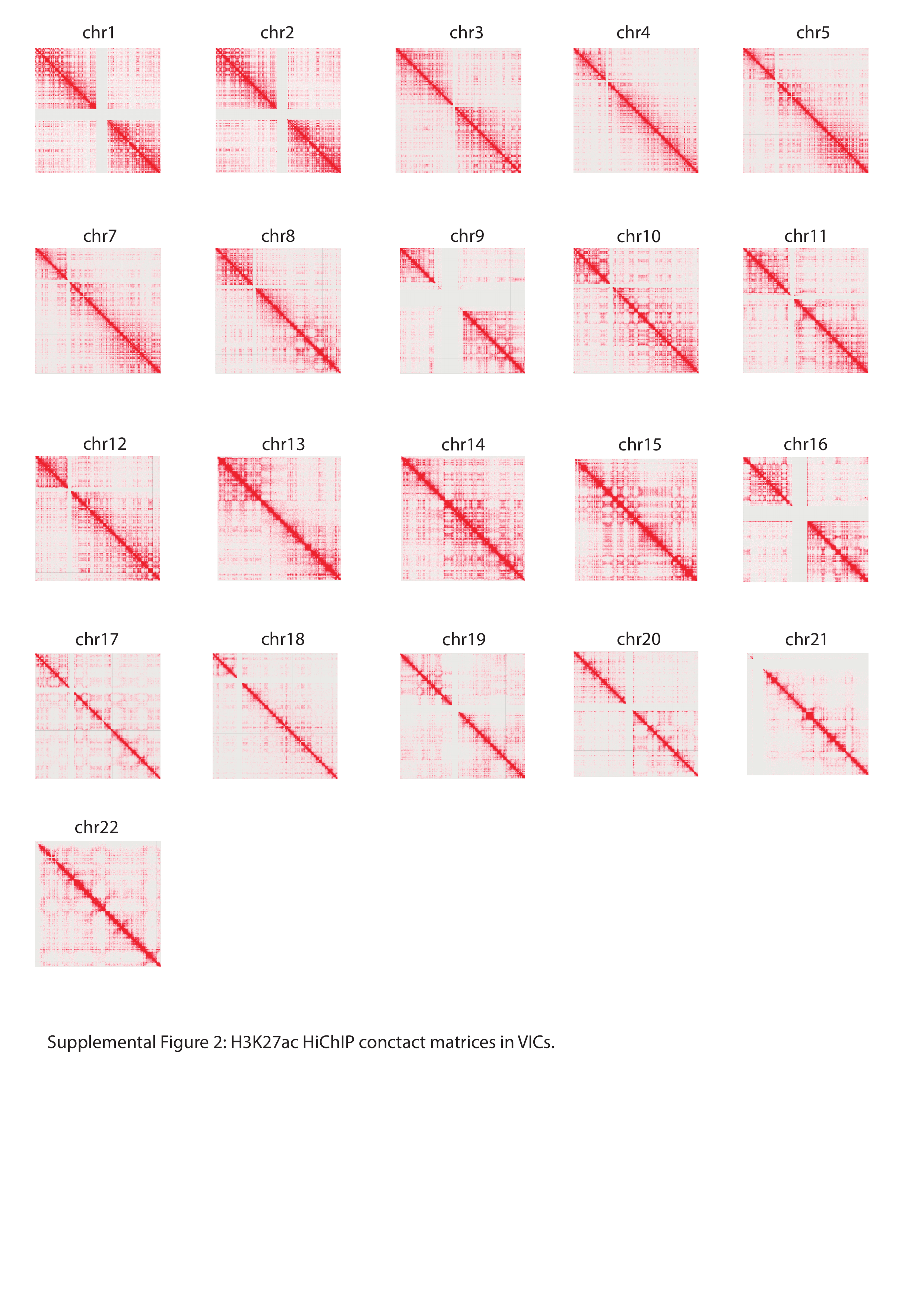

Supplement: S2 Fig — (TIFF) [file pgen.1010010.s002.tiff]

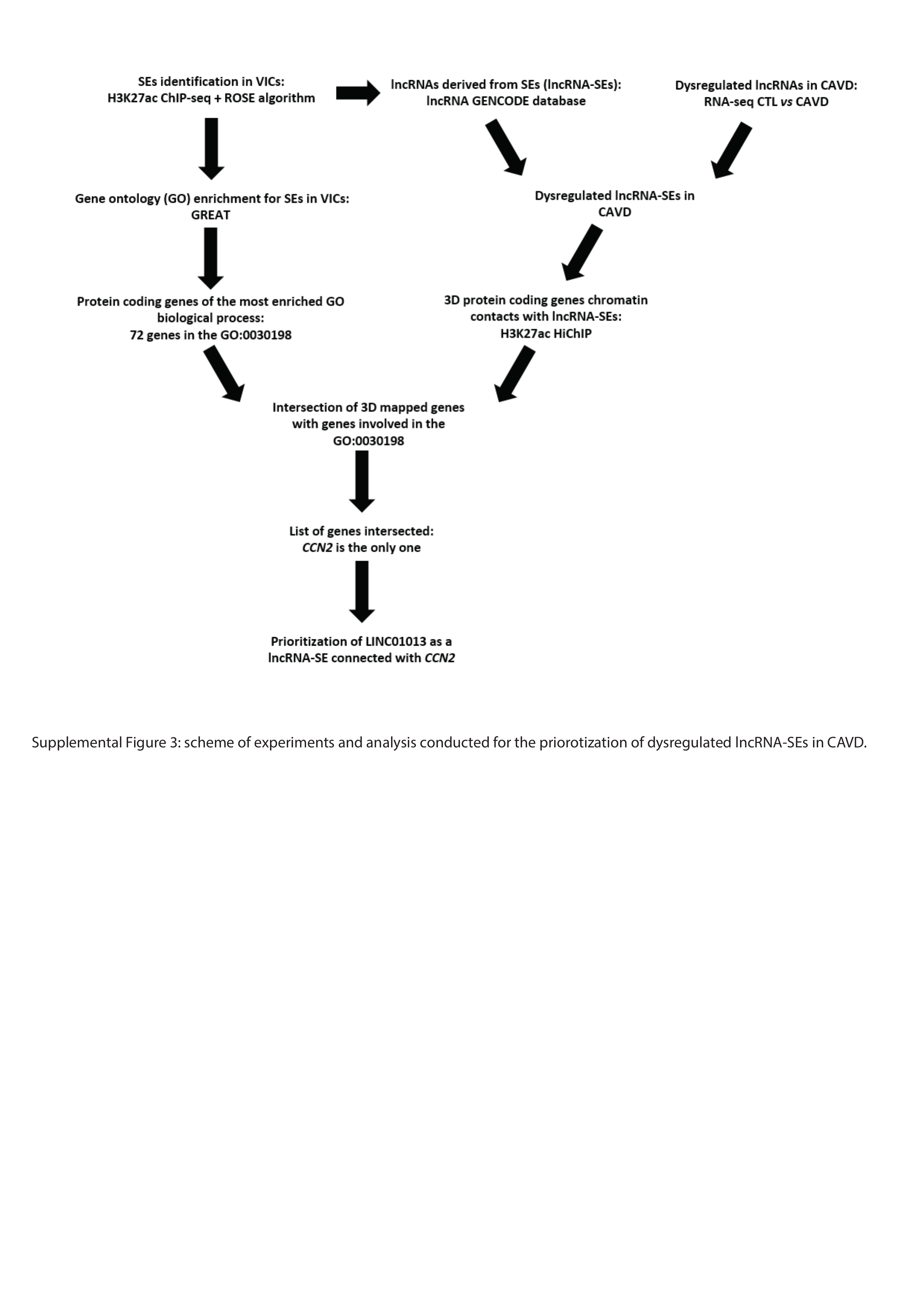

Supplement: S3 Fig — (TIFF) [file pgen.1010010.s003.tiff]

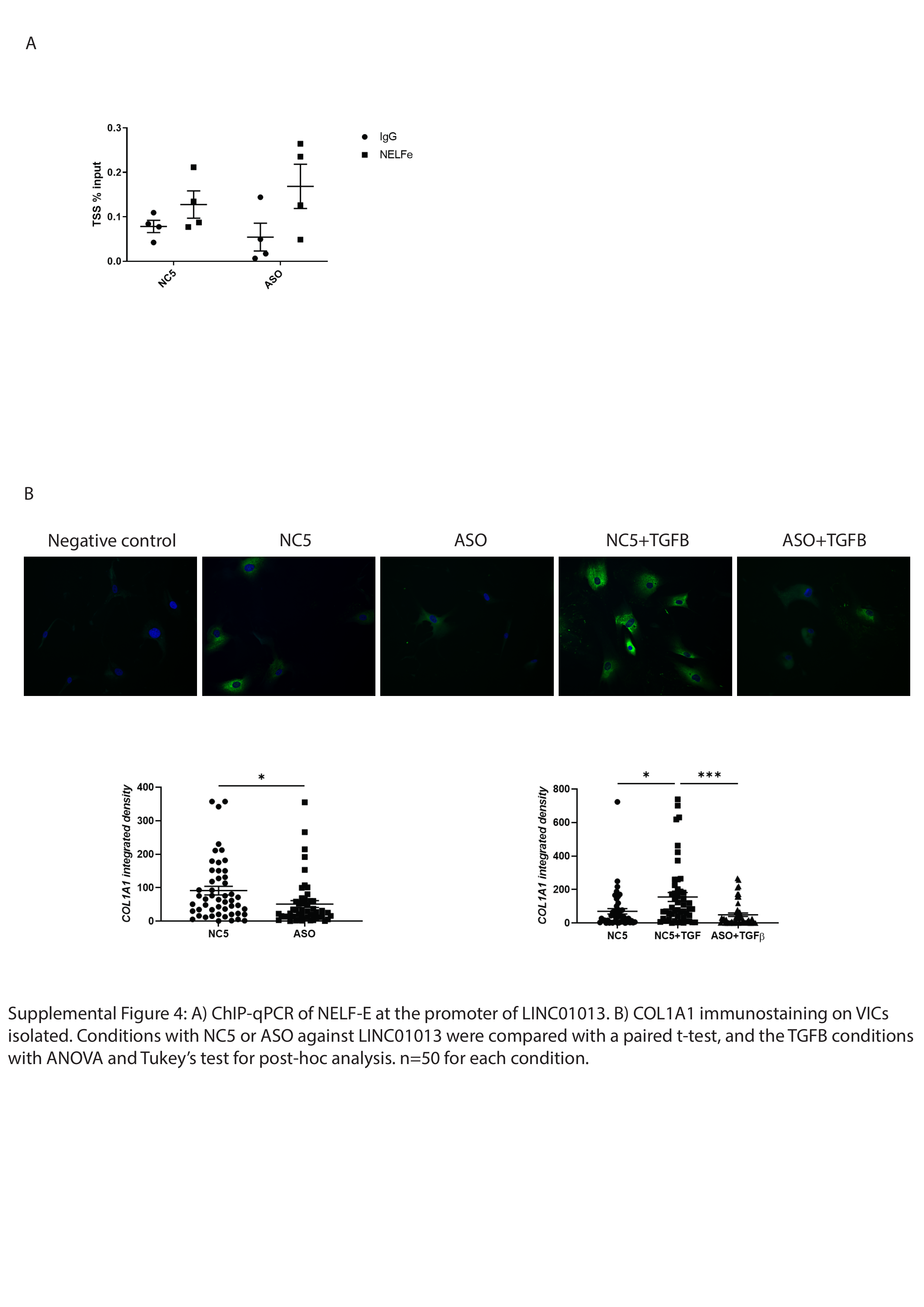

Supplement: S4 Fig — A) ChIP-qPCR of NELF-E at the promoter of LINC01013. B) COL1A1 immunostaining on VICs. Conditions with NC5 or ASO against LINC01013 were compared with a paired t-test, and the TGFB conditions with ANOVA and Tukey’s test for post-hoc analysis. n = 50 for each condition. (TIFF) [file pgen.1010010.s004.tiff]
