# Supplementary material for: Genome-wide chromatin contacts of super-enhancer-associated lncRNA identify LINC01013 as a regulator of fibrosis in the aortic valve
Source: PLoS Genet. 2022 Jan 18;18(1):e1010010. doi: 10.1371/journal.pgen.1010010 (PMC8797204; doi:10.1371/journal.pgen.1010010)
Supplement: Attachment — Submitted filename: Response_R1_PM.docx [file pgen.1010010.s013.docx]

Reviewer #1: In their manuscript, Chignon et al. seek to determine if super enhancer (SE)-derived lncRNA RECAST regulates fibrosis in the aortic valve. Their study uses an impressive array of molecular biology techniques, next-generation sequencing, and bioinformatic analyses to identify a putative SE-derived lncRNA associated with calcific aortic valve disease. They identified RECAST as an SE-lncRNA that positively controls CCN2 expression by acting as a decoy for the NELF-E complex, leading to fibrosis. Although of interest, the manuscript should be edited for clarity and to provide sufficient background to lead the reader logically through the experiments. In addition, the studies supporting RECAST sequestration of NELF_E should be linked to functional studies.

Main comments
1) In the introduction, lncRNAs are presented as fine-tuners of gene expression and as such could be targeted to reduce fibrosis. It would be appropriate to introduce the successes or failure of conventional therapies targeting fibrotic pathways in cardiovascular disease before discussing a potentially new therapeutic target. As one of the major findings concerns CCN2, it would be helpful to introduce CCN2 in the introduction as well.

*Thank you for your suggestions. We have expanded the introduction section in order to introduce the concept related to fibrosis (page 4, lines 16-17; page 5, lines 5-7).*

2) The two different models used in the manuscript (VIC, AV from patients) need to be clearly introduced and it should be noted which model data comes from. As written, it is often unclear which of the two models the data comes from.

*We have highlighted in the manuscript if cells (VICs) or tissues (AVs from patients) were used for the experiments. In addition, we have added a scheme which details each model used (Supplemental Figure 1 cited at page 5, line 10).*

3) A clear statement of the objective/hypothesis is needed in the first paragraph of the result section, in order to help understand the progression of the experiments pertaining to Figure 1. For example, what is used as a control for the enrichment in SE in VICs is shown in Figure 1? If an enrichment is observed, what is base value for this enrichment?

*Thank you for your suggestion. We have added statement to clearly state the hypothesis (page 5, lines 15-18). We used the GREAT tool (PMID: 20436461) for enrichment analysis which performs a binomial test to identify enrichments from the deviations of a theoretically expected distribution of observations to control for false positives: we have added these details in the Methods section (page 19, lines 15-16).*

4) Provide context for using the ROSE algorithm. Is it to identify the SE or to rank the SE? Can the findings be replicated using other algorithms such as MARGE or ChromHMM?

*The ROSE algorithm, which has been developed by the team who characterized SEs for the first time (PMID: 23582322), is a widely used method to identify SEs. First, the algorithm performs a ranking of strong and close (stitched enhancers) H3K27ac peaks. Then, peaks are plotted on a scatter plot and the inflection point on the graph (the point where the slope is 1) is then used to identify super-enhancers from peaks ranked upper the inflection point. We used HOMER to confirm data obtained by ROSE, and we found that among the 958 SEs identified by HOMER, 83.3% overlapped with the SEs identified by ROSE including among them, the SE at the LINC01013/RECAST locus. We have added those details in the manuscript page 5, lines 19-22 and page 6, lines 1-2, and a new Supplemental Table 2 for the SEs identified by HOMER.*

5) Figure 1:
a. Why switch from Box Plot to Violin Plot? Is Figure 1E a different representation of Fig 1F?
b. R-SEs are introduced in Figure 1, what is it besides a ‘regular SE’?

*We have modified the Figure 1F for a Box Plot in order to harmonize with the Figure 1E. R-SE refers to a “regular SE” which is a SE from which no lncRNA is transcribed. Point described at page 6, line 14.*

6) In Figure 2, why present data from chromosome 6? It only becomes clear later that CCN2 and RECAST are located on chr.6. My general issue with this sequence of figures/data is that it is presented as an unbiased systemic approach, but the data clearly suggests that the authors had already identified RECAST and are using the experiments presented in Figure 1 and 2 to justify the investigation. The authors should be transparent about the sequence of experiment leading to the discovery of RECAST.

*Thank you for raising this point. We have chosen the chromosome 6 because the framework used in this study prioritized LINC01013 and CCN2 located in this chromosome. We have added the new Supplemental Figure 2 to present all the matrices for the other chromosomes. In addition, we have added another new Supplemental Figure 3 to clarify the prioritization of LINC01013/RECAST and CCN2: our method was based on a holistic and unbiased approach integrating whole genome sequencing experiments.*

7) Figure 2B: It is unclear how the AV were prepared for RNA-sequencing. In addition, the authors report changes in lncRNA expression, but what of mRNA expression genes implicated in the fibrotic processes of interest to the study (CCN2, SMAD2/3, and TGFB1). Along those lines, Figure 2C is not-informative as it’s unreadable.

*We have added more information about the RNA sequencing experiment in the Methods section (page 23, lines 16-21). The main aim of our study was to prioritize one lncRNA involved in the pathobiology of CAVD. That is why we have focused on the differentially expressed lncRNAs between normal and mineralized aortic valves, and not on protein coding genes. However, we have used the RNA sequencing data for protein coding genes to study the putative role of the TGF beta-CCN2 axis during the disease: for that purpose we have inferred a protein-protein interaction network from mineralized aortic valves (details in the Methods section page 29, lines 9-12); we observed that the network was enriched for the TGF beta signaling (Figure 5B), and that CCN2 was highly connected with TGF beta signaling proteins like SMAD2/3 and COL1A1 (Figure 5A). Subsequent functional analyses have confirmed the importance of the LINC01013-TGF beta-CCN2 axis in the fibrotic response (Figure 6H and I). The Figure 2C represents the last step of our framework to prioritize a SE-lncRNA; described in the new Supplemental Figure 3. Figure 2C is the Venn diagram of 3D coding genes mapped by lncRNA-SE (coding genes connected by 3D interactions to lncRNA-SE), which overlapped with the genes included in the GO extracellular matrix organization (Venn diagram is a simple way to illustrate the findings). The diagram show that only CCN2 was overlapping (CCN2 has significant contact frequency with LINC01013 in HiChIP and site specific 3C).*

8) The authors claim, using Figure 2D and 3A, that the locus of RECAST interacts with the promoter region of CCN2, 180kb upstream. However, the HiC data provided shows many other possible connections. This should be discussed.

*The loops in Figure 2D represent all the significant chromatin contacts from the HiChIP experiment in this genomic region. There were significant contacts between a bin encompassing LINC01013/RECAST and a bin encompassing the promoter of CCN2 at a resolution of 1 kb. There were also, as you described, some other chromatin contacts at this locus, which is a frequent finding using HiC-derived techniques; many between different noncoding regions. We have added a sentence page 8, lines 10-13 to clarify this point. We confirmed these results at a higher resolution by using a site specific chromosome conformation capture (3C) experiment. The latter experiment showed chromatin contacts between LINC01013/RECAST and the promoter of CCN2, which was consistent with data obtained from the HiChIP.*

9) This reviewer is confused about Figure 3B.

*Figure 3B represents the short and the long transcripts of LINC01013 according to the lncRNA GENCODE v32 database. The arrows indicate the qPCR primers design for each transcript. We have modified the figure legend to clarify this point.*

10) In Figure 3C, what is the rational for performing an adjustment for sex and age? Is there a sex and age component to RECAST expression? If so, please report.
a. In addition, is table 1 a supplemental table or will it be included in the manuscript?
b. Please report the statistical analysis used to perform the adjustment.

*Sex (male) and age are associated with the risk of aortic stenosis and could be confounding variables also associated with the expression of LINC01013/RECAST. For that purpose, we thus performed a multivariate analysis adjusted for sex and age to measure the expression of LINC01013/RECAST during the disease independently of sex and age. We have added some these details in the Methods section (page 30, line 16) and in the result section we now include the non-adjusted p value (P<0.0001) (page 9, line 12). The Table 1 is not a part of the supplemental tables and it will be included in the manuscript.*

11) Figure 3D, are the IgG data matched per sample or were a defined set of samples chosen for the IgG vs the H3K27ac procedure? Why are there fewer samples in the control H3K27ac compared to other conditions?

*The IgG were used as a negative control for each chromatin immunoprecipitation experiment. Control aortic valves are collected from heart transplant donors. Hence, control aortic valves are more difficult to collect compared to CAVD tissues.*

12) The authors should perform RNA-FISH in combination with IF, as well as DNA-FISH in combination with RNA-FISH to show colocalization of CCN2, RECAST, RECAST-SE-locus and NEFLE.

*DNA-FISH and 3C-based techniques rely on different concepts. Nevertheless, in several works the two techniques (DNA-FISH and 3C and its derivatives) provided largely concordant results (reviewed in PMID:* *27760553). In the present work, we opted for two complementary approaches: HiChIP and site specific 3C. HiChIP and 3C provide a high-resolution 3D map. Both approaches provided consistent results with regard to the interaction of SE-lncRNA (LINC01013) and CCN2. The implementation of DNA-FISH is labour-intensive and will require several months of work. Considering the time constraint imposed by the journal for the resubmission, DNA-FISH was not possible to implement. However, in line with a large body of literature, we believe that 3C based techniques provide at least a reliable assessment of chromatin conformation. Nonetheless, we have added a statement in the limitation section of the discussion: «* *The frequency of genome interactions were evaluated by 3C-based technique. Whether the assessment of 3D genome interaction at LINC01013 using distance-based technique such as DNA-FISH would provide similar results remains to be investigated.» (page 15 last paragraph).*

13) Figure 4A should include appropriate controls for cytoplasmic and nuclear RNAs.

*We have added in Figure 4A U6 and HPRT as controls respectively for nuclear and cytoplasmic compartments.*

14) Figures 4 and 6 would benefit from either functional data and/or collagen immuno-staining to show effects of the RECAST ASO on whole cells, not just cell lysate.

*We performed an immunostaining for* COL1A1 *in VICs in culture (see the Methods section, page 29 and 30). We have added the results the new Suppl. Figure 4B and discussed at page 12, lines 2-4. These data are consistent with the effects of the ASO against LINC01013/RECAST on COL1A we previously documented in the Figure 6A, 6B, 6F and 6G.*

15) More mechanistic data is needed to show that NELF-E is sequestered from the promoter of CCN2 by RECAST. If the model presented in Figure 6J is true, ChIP of NELF-E in cells treated with the RECAST ASO should reveal stronger attachment of NELF-E to the promoter region of CCN2, compared to control cells where RECAST sequesters NELF-E.

*Yes. After demonstrating that LINC01013/RECAST interacted with NELFE (Figure 4E), we demonstrated that its knockdown with the ASO is associated with an increase of the recruitment of NELF-E around the transcription start site (TSS) of CCN2 (Figure 4F). These data are consistent with the mechanisms known about the ability of some lncRNAs to sequester NELF-E and restrain its recruitment at the TSS of protein coding genes (PMID: 25263592 and 31624086).*

Reviewer #2: 1. A Google Scholar search for LINC01013 shows there are already several manuscripts about this lncRNA, including one that gave it a different name, Aerrie ([https://www.ncbi.nlm.nih.gov/pmc/articles/PMC7829583/](https://can01.safelinks.protection.outlook.com/?url=https%3A%2F%2Fwww.ncbi.nlm.nih.gov%2Fpmc%2Farticles%2FPMC7829583%2F&data=04%7C01%7Carnaud.chignon%40criucpq.ulaval.ca%7C2998a77c721e490a7fac08d91aba1d4c%7C56778bd56a3f4bd3a26593163e4d5bfe%7C1%7C0%7C637570208987514008%7CUnknown%7CTWFpbGZsb3d8eyJWIjoiMC4wLjAwMDAiLCJQIjoiV2luMzIiLCJBTiI6Ik1haWwiLCJXVCI6Mn0%3D%7C1000&sdata=9vftAYpw%2FbgMjL7tOAumnujGad%2B6ov4q179phU%2FHX74%3D&reserved=0) ). The results of the new study should be discussed in the context of the previous research, and it is better to use the name already given in the literature (and to make sure its registered at genenames.org).

*Regarding to your point, we have replaced RECAST by LINC01013 in the whole manuscript.*

2. Is RECAST conserved in mouse? Is the SE it is transcribed from conserved in mouse? If yes – is it active in similar cells? These questions are important for future studies of RECAST in vivo and should be discussed.

*This is a good point. According to the NONCODE database, LINC01013/RECAST is only expressed in human. We have clarified this point in the manuscript page 8, line 10. We have discussed this point in the Limitations section page 16, lines 1-3.*

3. Does knockdown of RECAST affect levels of NELF-E recruited to the SE in the RECAST promoter region?

*Following your suggestion, we have measured the recruitment of NELF-E at the promoter of LINC01013/RECAST. We observed a poor enrichment of NELF-E at this region, and no impact of the knockdown of LINC01013/RECAST on its recruitment. We have added these results in the Supplemental Figure 4A.*

4. Since a main argument of the manuscript is that RECAST affects Pol2 pausing at the CCN2 promoter, the authors should use ChIP-seq or Cut&Run to measure Pol2 pausing in the control and the ASO-treated cells. Cut&Run does not require a large number of cells, so it should be feasible. This will allow the authors to measure pausing directly, and will provide much more convincing evidence than the presented qPCR data on no change in Pol2 levels and increased in Ser5P-modified Pol2.

*This is a good point. Pol2 pausing is a complex process and a clear demonstration of pause would need to set up specific conditions and measure the pausing index. As suggested, we have performed a ChIP-seq for Pol2 in VICs (with and without ASO), but we experienced difficulties with the small amount of cells and efficacy of the IP. Please note, since the start of the COVID pandemic the supply of primary cells has been slowed down considerably for logistic reasons out of our control and thus some experiments needing a large amount of difficult-to-grow primary cells are more laborious to perform. Considering the time constraints for the resubmission, it was not possible to redo the experiment and to include Pol2 ChIP-seq data. We have thus modified the wording throughout the text to strictly limit our conclusion to observations: impact of LINC01013 on the recruitment of NELFE and Ser5P-Pol2. We have also included this point as limitation in the discussion section (page 16, lines 3-6): «* *In the present study, strong data militate for a role of LINC01013 on the expression of CCN2 by modifying the recruitment of NELFE, an important regulator of RNAPII. However, further work is needed to assess whether LINC01013 affects the processivity of RNAPII.»*

Minor comments:
1. Abstract “located at ~180 kb” – its not clear what this refers to - should be “at ~180 kb upstread/downstream of CCN2”.

*We have modified in the abstract: «...* *located at ~180 kb upstream».*

2. Figure 1A – many of the categories shown are very similar/redundant. Can the authors filter out categories that overlap by >X% of the genes?

*Each category refers to a specific gene ontology (GO) number according to the Gene Ontology database (for example GO:0030198 for the GO “extracellular matrix organization”). We agree that GO is somewhat redundant. However, excluding some GO would be largely arbitrary and would not modify the conclusion. We have thus elected to keep the GO results as provided from the analysis pipeline from GO.*

3. “R-SE” should be defined in the legend of Fig. 1C

*Thank you, we have defined “R-SE” as “regular SE, and described in the figure legend: «(SEs for which no lncRNA is transcribed are defined as regular SE (R-SE))»*

4. Fig. 2A – the authors should show the positions of RECAST and CCN2 also on the vertical axis. Also, it should be indicated in the legend that these are HiChIP data (as this presentation is typically used for HiC).

*We agree. We have added the names of LINC01013/RECAST and CCN2 on the vertical axis.*

5. Its not clear what Fig. 3B is showing – just the position of the primers? Where is the evidence on which isoform is expressed? Anyhow, this should be a supplementary panel.

*Figure 3B represents the position of the primers. We have added some details in the figure legend: «* *Regions selected for the design of primers targeting the long or the short transcripts of LINC01013 (3 or 2 exons).» We did not detect the short transcript by RT-qPCR as detailed page 9, lines 6-7.*

6. Fig. 4A – the data used for normalization (cytoplasmic/nuclear RNAs) should be shown for scale.

*This is a good point, we have added controls in Figure 4A (U6 and HPRT). We normalized each compartment for the total RNA level in cells. We have added these details in the methods section page 27, lines 4-6.*

7. Page 5 “1085 SE (Suppl. Table 1), which were highly enriched in gene ontology” – its is not clear how SEs can be enriched in gene ontology. Where these SEs first assigned to genes and the genes were tested for enrichment? How many genes? How were these assigned? This should be clarified in the results section.

*We used GREAT (PMID: 20436461) to perform the enrichment analysis of SEs. GREAT provides enrichment test by mapping genomic regions to protein coding genes (from which enrichments are performed). It performs a binomial test to identify enrichments from the deviations of a theoretically expected distribution of observations to control for false positives. We have added details in the method section (page 19, lines 18-19) to clarify this point.*

8. Page 6 “We identified 324 lncRNA-SE” – does this refer to the number of lncRNAs or the number of SEs? Assuming a SE can overlap more than 1 lncRNA and perhaps vice versa, this should be clarified.

*We have included this statement: “By using an extensive annotation of lncRNAs (GENCODE version 32) that was intersected with the SE dataset, we identified 324 SE overlapping with one lncRNA” (in the present case, each SE overlapped with only one lncRNA) (page 6, lines 10-12).*
