# Supplementary material for: Genome-wide chromatin contacts of super-enhancer-associated lncRNA identify LINC01013 as a regulator of fibrosis in the aortic valve
Source: PLoS Genet. 2022 Jan 18;18(1):e1010010. doi: 10.1371/journal.pgen.1010010 (PMC8797204; doi:10.1371/journal.pgen.1010010)
Supplement: Attachment — Submitted filename: Response_11112021_R2_v2_DA_PM.docx [file pgen.1010010.s014.docx]

**To the editor and reviewers:**

We thank the reviewers/editors for their time and constructive comments. Their inputs have clearly improved the accuracy of our statements and the clarity of our manuscript. The references cited in this document are located at the end.

**Comments to the Authors:**

**Reviewer #1:** The authors of ‘Genome-Wide Chromatin Contacts of Super-EnhancerAssociated lncRNA Identify LINC01013 as a Regulator of Fibrosis in the Aortic Valve’ have successfully responded to many of the issues brought up by the reviewers. They have improved their manuscript for clarity and (scientific) depth. For example, the addition of secondary (in silico) approaches, such as HOMER, extra supporting information, more detailed methodologies and further explanation how certain enrichment analysis are used have boosted the overall quality of the paper.

There is one issue that still remains: In the graphical representation of the proposed molecular mechanism, the authors propose an aggregate of Linc01013, NELF-E, RNAPII and CCN2 upon treatment with TGFbeta. This putative interaction is, as the authors describe in their rebuttal, shown by HiChIP and 3C. Indeed, HiChIP can be used to determine protein-directe genome architecture and 3C to capture the 3D conformation of the chromosome. However, neither of these techniques (as far as this reviewer is aware) can pinpoint RNA presence at the DNA level. However, it would be of high relevance if the authors can show the interaction on a more granular level and the RNA-transcripts is present at this particular genomic location. As the authors claim that the implementation of DNA-FISH is labor-intensive this can be shown in a variety of other ways:

- RNA-FISH for Linc01013 + IF for NELF-E to show colocalization of the two molecules in the cell.

- ChIRP-PCR to show localization of Linc01013 to the genomic locus presented in Figure 6J (ChIRP-Seq to see where else NELF-E and Linc01013 could work together would be best, but likely out of scope of this project).

- Enhance pull-down data to further validate the interaction between NELF-E and Linc01013 with in silico evidence (catRAPID or alike), followed by (simple) mutation in vitro studies on the RNA/protein interaction domain (overexpression followed by RIP-PCR).

**Response to reviewer #1:**

Thank you for raising these points. Our study demonstrated that the transcript *LINC01013* affected the expression of *CCN2* by acting as a molecular decoy for NELFE, which kept NELFE away from chromatin. A series of experiments (3C (figure 3A), RIP (figure 4E), ChIP NELFE (figure 4F) ) was designed to evaluate the importance of this mechanism. By regulating its neighboring gene expression (*CCN2*) *LINC01013 is* acting as a molecular sink for NELFE.

As this reviewer suggested, it would be interesting to visualize the proximity of *LINC01013* with the *CCN2* locus. However, all the mentioned experiments are time-consuming. One of the technical limitation with RNA-FISH for native *LINC01013* and IF for NELFE is the low expression of *LINC01013* in the VIC cells, a difficult-to-grow primary cell with a low replicative capacity. Considering that the journal allowed 30 days for resubmission, it was not possible to optimize the conditions for the hybridization efficiency, the accessibility and visibility of fluorescent probes within the nucleus. This series of experiments would take several months to perform in primary cells such as VICs. Nonetheless, we would like to point out that we demonstrated the proximity of the two loci (*LINC01013* and *CCN2*) by using well-validated approaches such as HiChIP and locus specific 3C with quantitative PCR.

We demonstrated the interaction between NELFE and native *LINC01013* transcript by using RIP experiments in primary VIC cells*.* As suggested by this reviewer, we used computational prediction tool as an additional approach to assess the interaction between these two molecular actors. We used catRAPID algorithm to estimate the binding propensity of NEFLE-*LINC01013* pair and to detect RNA motifs as well as protein RNA binding domains involved in the interaction [1]. By using catRAPID, we were unable to predict domains, which would involve the interaction of *LINC01013* with NELFE. To evaluate catRAPID’s performance on interactions between NELFE and lncRNAs, we performed the same approach using lncRNAs ARC and RAIN as positive controls. Previous reports showed an interaction between these two lncRNAs with NEFLE [2, 3]. Of note, catRAPID did not predict an interaction between ARC and NELFE, whereas it was not possible to conclude for RAIN as the sequence of the isoform in the manuscript has not been made public. The structure of NELFE is well described in the literature [4]. NELFE, mediates the binding of the NELF complex to nascent RNAs through its [RNA recognition motif](https://www.sciencedirect.com/topics/biochemistry-genetics-and-molecular-biology/rna-recognition-motif) (RRM) [5]. Therefore, it is likely that the interaction between *LINC01013* with NELFE is mediated by the RRM. To validate this hypothesis, it would be interesting to perform RIP experiments with plasmids encoding truncated forms of NELFE, and especially a mutant of NELFE without the RRM domain. After a search, these mutants are not commercially available. Again, performing this series of experiments and generate the different constructs will take several months, which is beyond the time-frame of 30 days allowed by the journal for this revision, and is more likely an endeavour for follow-up studies.

**Reviewer #2:** The authors have addressed my comments from the previous round of review in a largely satisfactory manner, and have improved the manuscript. They should nevertheless, discuss their results in the context of the other studies on LINC01013, and mention the fact that HiCHIP data are presented in Fig. 2A in the figure legend, both points as requested in the previous round of review. I can then recommend publication in PLoS Genetics.

**Response to reviewer #2:**

Thank you for your suggestions and encouraging comments. As mentioned, we included a more detailed description of the literature about LINC01013 in the discussion section and two references (please see from page 14, paragraph 2 to page 15 paragraph 1). Please see novel Figure legend 2A at page 33, paragraph 2 of the revised manuscript. Now, you will find that the interaction matrices were generated using the H3K27ac HiChIP experiments in VICS.

**Have all data underlying the figures and results presented in the manuscript been provided?**
Large-scale datasets should be made available via a public repository as described in the *PLOS Genetics* [data availability policy](https://can01.safelinks.protection.outlook.com/?url=http%3A%2F%2Fjournals.plos.org%2Fplosgenetics%2Fs%2Fdata-availability&data=04%7C01%7Cpatrick.mathieu%40fmed.ulaval.ca%7Ce34a2dcc185a4f2b042608d98fe6a82b%7C56778bd56a3f4bd3a26593163e4d5bfe%7C1%7C0%7C637699043095114301%7CUnknown%7CTWFpbGZsb3d8eyJWIjoiMC4wLjAwMDAiLCJQIjoiV2luMzIiLCJBTiI6Ik1haWwiLCJXVCI6Mn0%3D%7C1000&sdata=RAWoYOA3lXb7BA%2Bea9qLCyP%2BqwF1qcO7LLPI9ivBDz0%3D&reserved=0), and numerical data that underlies graphs or summary statistics should be provided in spreadsheet form as supporting information.

Reviewer #1: Yes

Reviewer #2: Yes

PLOS authors have the option to publish the peer review history of their article ([what does this mean?](https://can01.safelinks.protection.outlook.com/?url=https%3A%2F%2Fjournals.plos.org%2Fplosgenetics%2Fs%2Feditorial-and-peer-review-process%23loc-peer-review-history&data=04%7C01%7Cpatrick.mathieu%40fmed.ulaval.ca%7Ce34a2dcc185a4f2b042608d98fe6a82b%7C56778bd56a3f4bd3a26593163e4d5bfe%7C1%7C0%7C637699043095114301%7CUnknown%7CTWFpbGZsb3d8eyJWIjoiMC4wLjAwMDAiLCJQIjoiV2luMzIiLCJBTiI6Ik1haWwiLCJXVCI6Mn0%3D%7C1000&sdata=YJKmKjQqGrAQpthEIl0gdQiQatBTl1G9PrLwxoYFX1Y%3D&reserved=0)). If published, this will include your full peer review and any attached files.


If you choose “no”, your identity will remain anonymous but your review may still be made public.


**Do you want your identity to be public for this peer review?** For information about this choice, including consent withdrawal, please see our [Privacy Policy](https://can01.safelinks.protection.outlook.com/?url=https%3A%2F%2Fwww.plos.org%2Fprivacy-policy&data=04%7C01%7Cpatrick.mathieu%40fmed.ulaval.ca%7Ce34a2dcc185a4f2b042608d98fe6a82b%7C56778bd56a3f4bd3a26593163e4d5bfe%7C1%7C0%7C637699043095114301%7CUnknown%7CTWFpbGZsb3d8eyJWIjoiMC4wLjAwMDAiLCJQIjoiV2luMzIiLCJBTiI6Ik1haWwiLCJXVCI6Mn0%3D%7C1000&sdata=56rFMfMnKT7QcAi2vNVrI%2Fj4n0ftZuBl2rw2FgGsJ4E%3D&reserved=0).

Reviewer #1: No

Reviewer #2: No

References:

1.	Agostini F, Zanzoni A, Klus P, Marchese D, Cirillo D, Tartaglia GG (2013) catRAPID omics: a web server for large-scale prediction of protein–RNA interactions. Bioinformatics 29:2928–2930

2.	Schaukowitch K, Joo J-Y, Liu X, Watts JK, Martinez C, Kim T-K (2014) Enhancer RNA facilitates NELF release from immediate early genes. Mol Cell 56:29–42

3.	Rossi T, Pistoni M, Sancisi V, Gobbi G, Torricelli F, Donati B, Ribisi S, Gugnoni M, Ciarrocchi A (2020) RAIN Is a Novel Enhancer-Associated lncRNA That Controls RUNX2 Expression and Promotes Breast and Thyroid Cancer. Mol Cancer Res 18:140–152

4.	Vos SM, Farnung L, Urlaub H, Cramer P (2018) Structure of paused transcription complex Pol II–DSIF–NELF. Nature 560:601

5.	Yamaguchi Y, Inukai N, Narita T, Wada T, Handa H (2002) Evidence that negative elongation factor represses transcription elongation through binding to a DRB sensitivity-inducing factor/RNA polymerase II complex and RNA. Mol Cell Biol 22:2918–2927
