# Supplementary material for: Genome-wide chromatin contacts of super-enhancer-associated lncRNA identify LINC01013 as a regulator of fibrosis in the aortic valve
Source: PLoS Genet. 2022 Jan 18;18(1):e1010010. doi: 10.1371/journal.pgen.1010010 (PMC8797204; doi:10.1371/journal.pgen.1010010)
Supplement: Attachment — Submitted filename: response_to_reviews_13122021.docx [file pgen.1010010.s015.docx]

The editors would ask that you refer back to the comments of Reviewer 1 from the previous round of review (please note that there are no new reviewer comments to address, which is why no comments were pulled into the decision letter, and we apologise for the confusion):

"There is one issue that still remains: In the graphical representation of the proposed molecular mechanism, the authors propose an aggregate of Linc01013, NELF-E, RNAPII and CCN2 upon treatment with TGFbeta. This putative interaction is, as the authors describe in their rebuttal, shown by HiChIP and 3C. Indeed, HiChIP can be used to determine protein-directe genome architecture and 3C to capture the 3D conformation of the chromosome. However, neither of these techniques (as far as this reviewer is aware) can pinpoint RNA presence at the DNA level. However, it would be of high relevance if the authors can show the interaction on a more granular level and the RNA-transcripts is present at this particular genomic location. As the authors claim that the implementation of DNA-FISH is labor-intensive this can be shown in a variety of other ways: - RNA-FISH for Linc01013 + IF for NELF-E to show colocalization of the two molecules in the cell. - ChIRP-PCR to show localization of Linc01013 to the genomic locus presented in Figure 6J (ChIRP-Seq to see where else NELF-E and Linc01013 could work together would be best, but likely out of scope of this project). - Enhance pull-down data to further validate the interaction between NELF-E and Linc01013 with in silico evidence (catRAPID or alike), followed by (simple) mutation in vitro studies on the RNA/protein interaction domain (overexpression followed by RIP-PCR)."

The editors support the reviewer's point that direct RNA involvement in the mechanism has not been demonstrated, but they appreciate that these would be difficult experiments to perform. If you would like to address these issues experimentally, please do let us know. Otherwise, the editors would be happy for you to respond by acknowledging the issue in the text, and being more circumspect about the mechanism in the text and figures.

*A: Yes, we agree. As suggested by the editors we have modified Figure 6, legend of Figure 6 and the text in the discussion in order to acknowledge that the link between LINC01013 and DNA at the CCN2 locus is hypothetical. To this effect, we modified the figure 6: LINC01013 has been included on the exterior of the loop and we have included a dashed line to the locus to indicate that this process is possible, but remains to be demonstrated.*

*In Figure legend 6 panel J page 36 we have modified the caption to: «Cartoon illustrates the process whereby LINC01013 controls the expression of CCN2; dashed line is a hypothetical process as a direct interaction of LINC01013 with DNA at the CCN2 locus remains to be demonstrated.»*

*In the discussion page 16 limitations section, we have added the following sentences: «Though we showed that LINC01013 is a transcriptional regulator of CCN2 by acting as decoy factor for NELF-E, the present study did not investigate the possible direct interaction of LINC01013 with DNA at this locus. Further experiments are needed to address the interaction of LINC01013 with chromatin.»*
